# Ontogeny and Anatomy of the Dimorphic Pitchers of *Nepenthes rafflesiana* Jack

**DOI:** 10.3390/plants9111603

**Published:** 2020-11-18

**Authors:** Rachel Schwallier, Valeri van Wely, Mirna Baak, Rutger Vos, Bertie Joan van Heuven, Erik Smets, Rogier R. van Vugt, Barbara Gravendeel

**Affiliations:** 1 Naturalis Biodiversity Center, Darwinweg 2, 2333 CR Leiden, The Netherlands; valerivanwely@gmail.com (V.v.W.); rutger.Vos@naturalis.nl (R.V.); bertiejoan.vanheuven@naturalis.nl (B.J.v.H.); erik.smets@naturalis.nl (E.S.); barbara.gravendeel@naturalis.nl (B.G.); 2 Faculty of Science and Technology, University of Applied Sciences Leiden, Zernikedreef 11, 2333 CK Leiden, The Netherlands; baak.mirna@gmail.com; 3 Institute Biology Leiden, Leiden University, Sylviusweg 72, 2333 CC Leiden, The Netherlands; 4 Hortus Botanicus, Leiden University, Rapenburg 73, 2311 GJ Leiden, The Netherlands; vugt@hortusleiden.nl

**Keywords:** 3D geometric morphometrics (3DGM), carnivorous plants, development, landmark analysis, microstructure, pitcher plants

## Abstract

An enigmatic feature of tropical pitcher plants belonging to the genus *Nepenthes* is their dimorphic prey-capturing pitfall traps. In many species, the conspicuously shaped upper and lower pitchers grow from a swollen leaf tendril tip until finally opening as insect-alluring devices. Few have studied the ontogeny of these traps from an anatomical and quantitative morphological perspective. We investigated whether the anatomy and development of lower and upper type pitchers of *N. rafflesiana* differ or overlap in terms of 3D geometric morphology and microstructure progression and presence. We hypothesized that there is an overlap in the initial, but not all, developmental stages of the two pitcher types and that one pitcher type is suspended in development. We identified four important morphological changes of pitcher ontogeny and defined these as curvation, elongation, inflation and maturation phases. Pitcher length indicated progress through developmental phases, and we propose to use it as a tool for indication of developmental stage. Microstructure development coincided with the developmental phases defined. Additionally, we discovered a new anatomical feature of extrafloral nectariferous peristomal glands between the inner peristome ridges of upper and lower pitchers being hollow and analyze the chemistry of the sugars on the outside of these glands. Ontogenetic shape analysis indicated that upper and lower pitcher types develop with similar phase progression but have no directly overlapping morphology. This means that upper pitchers are not a derived state from lower pitchers. Independent developmental programs evolved to produce distinctly shaped upper and lower pitchers in *Nepenthes,* likely to exploit different food sources.

## 1. Introduction

The tropical carnivorous plant genus *Nepenthes* is characterized by one of the most striking morphological features of plants: an insect-luring pitfall trap. These traps, modified leaf tips called pitchers, are an adaptation to scarcity of nutrient resources and facilitate the capture of an abundant nitrogen alternative [1,2]. Throughout their distribution region in the Malay Archipelago, Australia, India, and Madagascar [3,4], the 140+ recognized species [5] attract insects, such as flies, ants, or termites, through visual lures [6,7], extrafloral nectar [8,9,10], and olfactory cues [11]. Food sources that are captured and retained via mechanisms, such as slippery ‘aquaplaning’ surfaces near the trap opening [12,13], viscous pitcher fluid [14,15,16], and waxy inner surfaces [17,18,19] are broken down through enzyme rich digestive fluids, bacteria, and microfauna [20,21,22,23,24]. Shape [10,25,26] and the pitchers’ various microstructures (i.e., digestive glands, extrafloral nectaries, cells that release wax, etc.) play a direct role in the capture and retention of prey [9,11,16,19,27,28]. Although the majority of species are believed to be prey generalists, capturing a wide variety of insects for their diet [1,29], several recent studies show that many species are quite specialized. Pitchers of *Nepenthes lowii* and *N. rajah*, for example, are distinctively shaped [10,30] for their diet specialization: they receive nutrient-rich feces from small mammals that sit over the trap opening while they feed on nectar produced in the lid. The single detritivore described, *Nepenthes ampullaria,* has an open-mouth that is largely reduced compared to other species and is associated with leaf-litter accumulations [31,32] and in the remarkable *N. gracilis,* large ants that are perched under the slippery waxed lid surface fall into its trap when a mere drop of rain falls from above [33].

Beyond the amazing morphological diversity of pitchers and microstructures within the genus, pitcher dimorphism of an individual plant is also quite striking [34,35]. The first mature pitcher type, or lower pitcher, is produced on or just above the forest floor during the earlier stages of mature plant growth, when plants are still self-supporting shrubs. With maturity, most species of *Nepenthes* become sprawling and climbing lianas that, in addition to lower pitchers, also produce upper pitchers that can be morphologically distinct as in *Nepenthes rafflesiana*. These upper pitchers are produced from leaf tips that occur higher up in the vegetation. Inventories of upper and lower pitchers show variation in diet [26,34,36], which suggests that having two functionally different pitchers could be an evolutionary advantage to capture a broader prey diversity, although contrasting prey from the differences in the entomofauna present in different strata is a challenge. In a comparative study of many data sources, Rembold et al. [34] found that the dimorphic pitchers of *N. gracilis, N. mirabilis*, and *N. madagascariensis* captured ground dwelling ants and that the upper pitchers additionally attracted flying, flower-visiting insects. Moran [36] found similar results in *N. rafflesiana*.

Despite the large differences in the two mature pitcher types of fully developed plants, little is known about them from an ontogenetic and morphometric perspective. There is an increasing interest and number of publications involving this genus, and, so far, no solid framework of pitcher development and its associated microstructures has been published. Although botanical morphology has historically been an important tool to taxonomists and ecologists, few botanical studies have employed the more recent technologies of 3D geometric-morphometric analysis yet [37,38] even though it has recently become an important tool in agriculture [39,40].

We hypothesize that there is an overlap in the initial, but not all, developmental stages of the two pitcher types, indicating that one pitcher type is suspended in development. We, therefore, investigated whether the anatomy and development of lower and upper type pitchers of *N. rafflesiana* differ or overlap in terms of (i) 3D geometric morphology and (ii) microstructure progression.

## 2. Results

### 2.1. Developmental Phases

**Figure 1:**
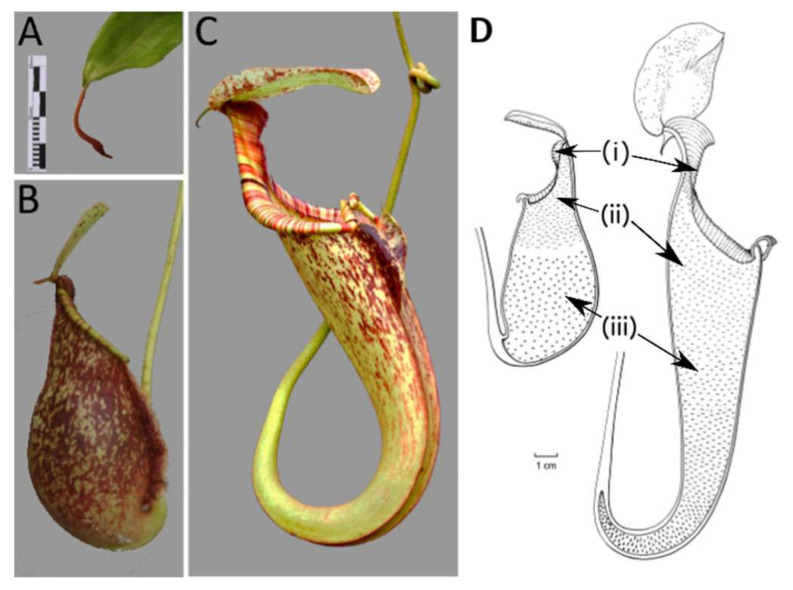
Pitcher dimorphism in *Nepenthes rafflesiana:* (**A**) The swollen tendril tip, (**B**) lower pitcher and (**C**) upper pitcher. Schematic longitudinal sections of *Nepenthes* (**D**) lower (left) and upper (right) pitcher, indicating (i) peristome, (ii) waxy glands, and (iii) digestive glands. Scale bar for (**A)**–(**C**) = 40 mm; scale bar for (**D**) = 1 cm. Photographs (**A**),(**B**) by Valeri van Wely, (**C**) by Rogier van Vugt. Illustration made by Esmée Winkel.

We identified and defined four distinct stadia based on clear morphological changes paralleled in upper and lower pitcher ontogeny. These were accordingly defined as the curvation phase, elongation phase, inflation phase and maturation phase. The first developmental phase begins when pitchers are first distinguishable as a swollen tendril tip, which extends from the midvein of the blade-like leaf (Figure 1A). This phase is further characterized by a strong curvature at the junction of tendril attachment to the pitcher. The flattened pitcher appearance changes in the inflation phase (Figures S1 and S2), when width increases, and length growth continues (Figures S3 and S4). Pitchers in the elongation phase increased considerably in length and depth (Figures S3 and S4). The coloration pattern of the pitcher becomes more apparent in this phase (Figures S1 and S2). The maturation phase is characterized by the lid opening. Phases identified during traditional morphometric analysis led our investigation of microstructure development through Scanning Electronic Microscopy (SEM) and Light Microscopy (LM).

### 2.2. Extrafloral Nectary and Peristomal Teeth

**Figure 2:**
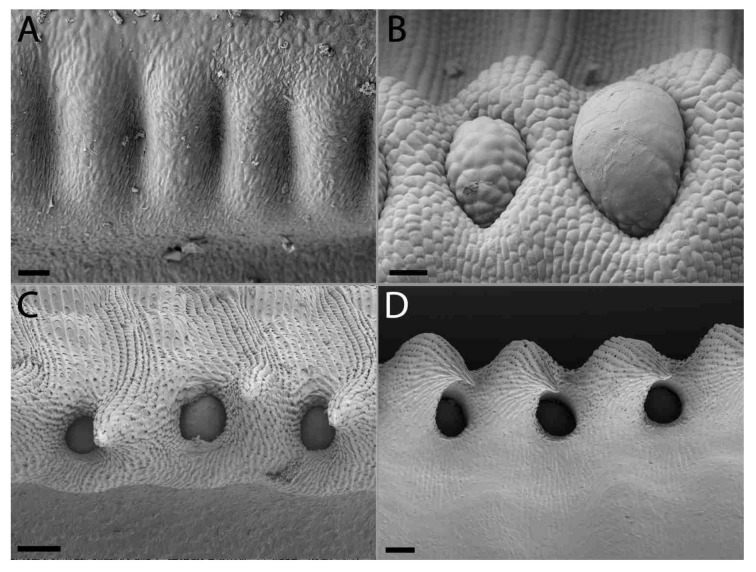
Peristomal gland development of lower *Nepenthes rafflesiana* pitchers. (**A**) Curvation phase—formation of peristome starts, glands absent. (**B**) Elongation phase—ridges clear, glands start developing at inner peristome. (**C**) Inflation phase—peristomal gland depressed in pits as peristomal teeth develop around them. (**D**) Maturation phase—Peristomal glands completely sunken into pits, flanked by fully developed peristomal teeth. (**A)**,(**B**) Scale bar = 20 µm and (**C)**,(**D**) scale bar = 100 µm.

**Figure 3:**
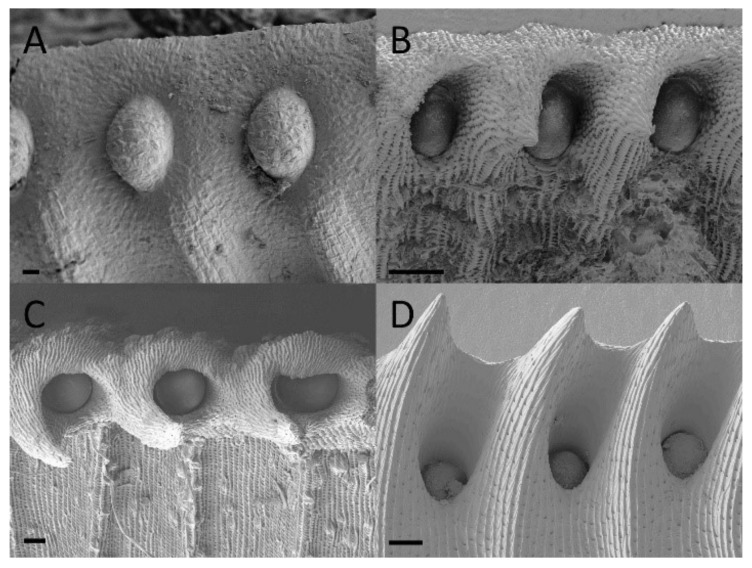
Peristomal gland development of upper *Nepenthes rafflesiana* pitchers. (**A**) Curvation phase—formation of peristome and glands. (**B**) Elongation phase—ridges and peristomal teeth clear, glands pitted in peristome. (**C**) Inflation phase—peristomal gland depressed in pits. (**D**) Maturation phase (photo from inside pitcher). Scale bar = 100 µm.

**Figure 4:**
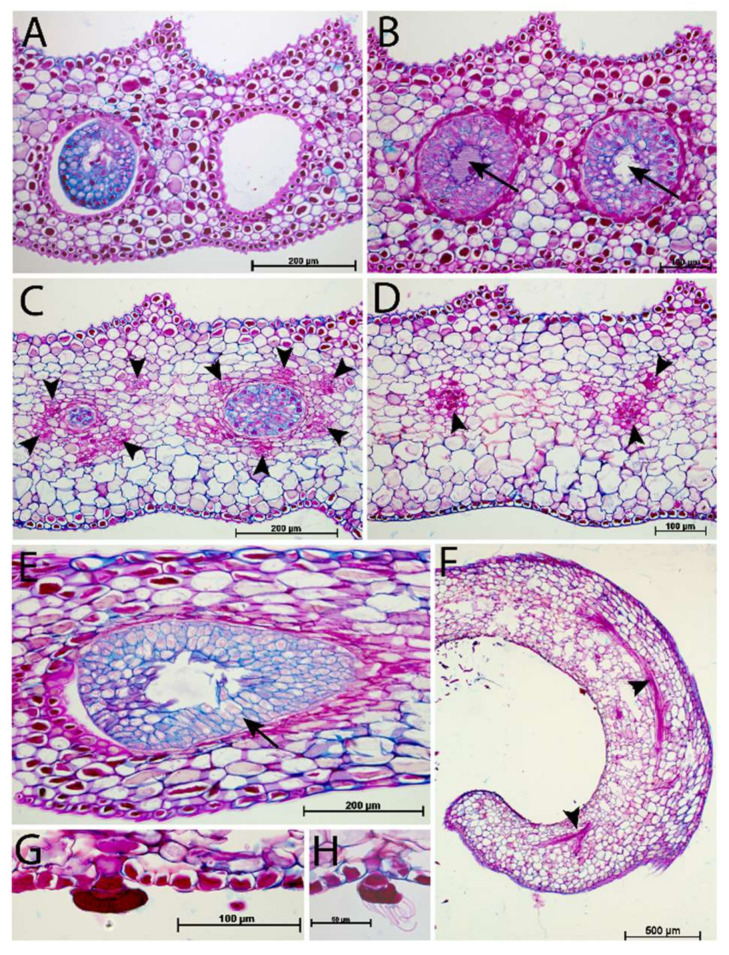
Light microscopic images of peristomal glands and vascular tissue from a mature *Nepenthes rafflesiana* pitcher. (**A**) Glands are present at the bottom of the pits between the peristomal teeth. (**B**) Cross sections through these glands show a hollow cavity (arrows) within peristomal glands. (**C**) Vascular tissue (arrowheads) in proximity to the deepest point of the peristomal glands. (**D**) Directly behind the glands, vascular tissue is also present. (**E**) Peristomal gland (oblique section). (**F**) Vascular tissue present in the outer arm of the peristome. (**G**) Second type of gland, from the underside of the outer arm of the peristome. (**H**) Fragments of collapsed cell. (**A**–**D**): Cross sections, (**E**–**H**): Longitudinal sections.

Progressive pitting of the peristomal nectaries characterizes the development of upper and lower pitchers of *N. rafflesiana*. Peristomal teeth begin as lateral ridges in the curvation phase and elongate through the remaining phases, eventually engulfing the peristomal glands in deep pockets between the mature curved teeth (Figure 2 and Figure 3). Light microscopy reveals glands of mature lower pitchers to have hollow cavities, surrounded by vascular tissue (Figure 4A–F). Peristomal fluid from on top of the peristome contained high amounts of sugars, indicated by a quick (within 1 min) color shift when mixed with Fehling’s solutions. NMR analysis of exudates from this gland further showed the presence of sugars (Figure S5). HNMR spectra of secretions from glands found on the underside of the lid also showed sugar presence (Figure S5). An additional stalked gland on the underside of the peristome was also visible with LM and was occasionally covered with structures reminiscent of leftover fragments (8n observations, all less than 1 cm in length/width) of a collapsed balloon-like structure (Figure 4H).

### 2.3. Digestive Glands

**Figure 5:**
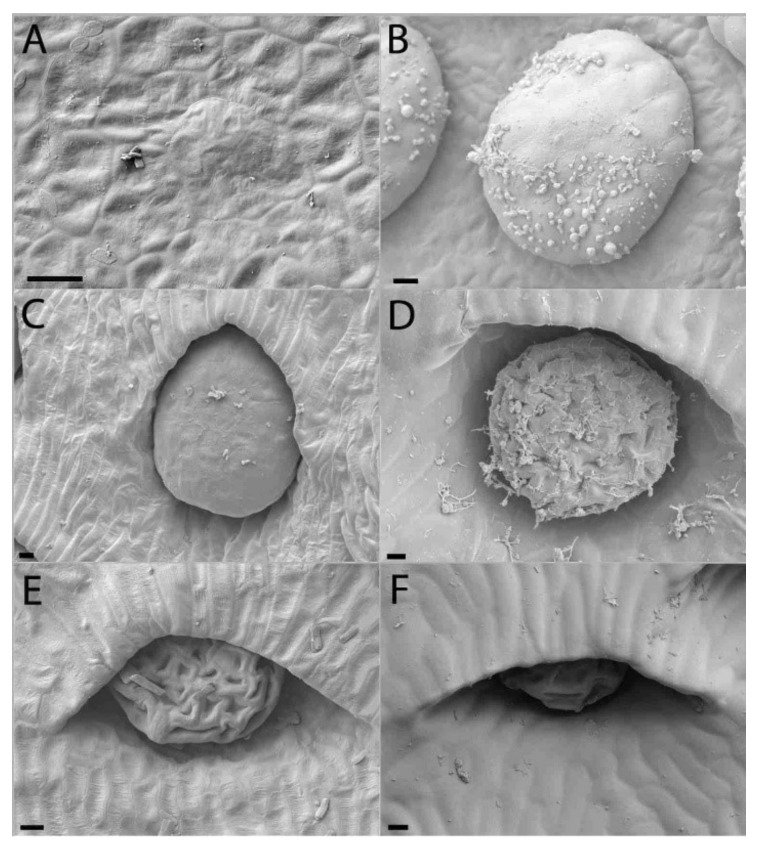
Digestive gland development of lower *Nepenthes rafflesiana* pitchers. (**A**) Curvation phase—small hump of cells visible. (**B**) Elongation phase—final size of gland reached and actively secreting. (**C**) Inflation phase—surrounding tissue changes, differentiates and envelops gland, which is even further exaggerated (**D**) higher on the pitcher. (**E**) Maturation phase—glands sunken into depression, and even further enveloped (**F**) higher up the pitcher. Scale bar = 10 µm.

**Figure 6:**
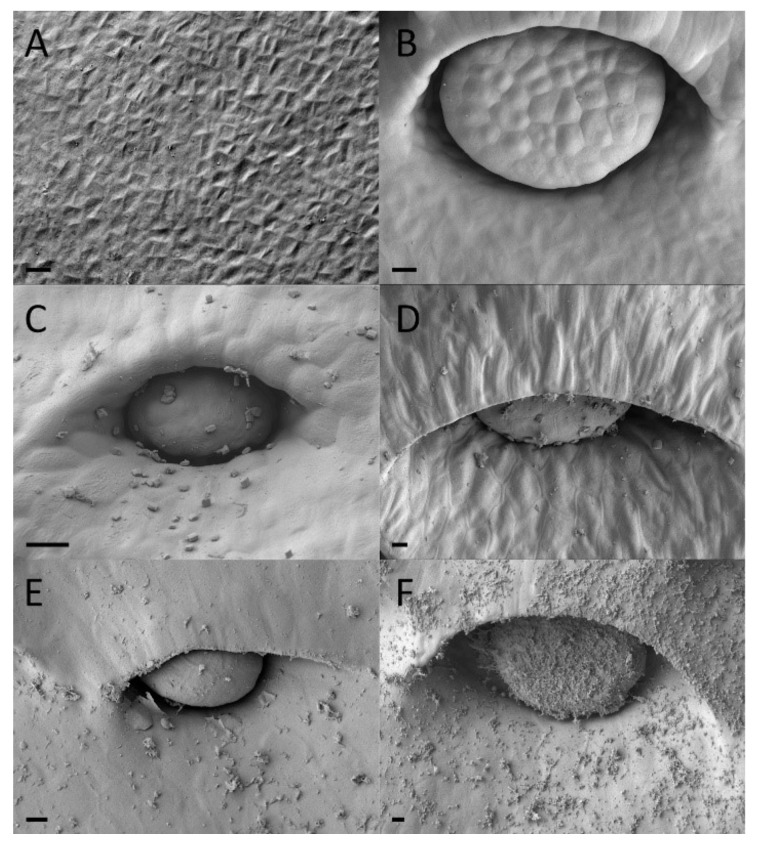
Digestive gland development of upper *Nepenthes rafflesiana* pitchers. (**A**) Curvation phase—faint cell formation visible. (**B**) Elongation phase—gland maximum size reached and slightly enveloped. (**C**) Inflation phase—gland more depressed into pit and enveloped considerably (**D**) higher up the pitcher. (**E**) Maturation phase—gland enveloped and even more so (**F**) higher up the pitcher. Scale bar = 10 µm.

Functional digestive glands of the dimorphic pitchers of *N. rafflesiana* mature early in development. Gland development begins in the curvation phase and its final size is quickly reached in the elongation phase (Figure 5 and Figure 6). Digestive glands actively secrete substance in the elongation phase of the lower pitcher as SEM showed substances on these glands that were completely absent from the surrounding tissue (Figure 5). Upper pitchers are at least active during the inflation phase as there is notable fluid inside the still unopened pitchers. Although gland size remains unchanged during inflation and maturation, the distance between the glands increases (Figures S6 and S7). Epidermal cells surrounding the glands differentiate to form an envelope structure over the top of the glands. These structures, or epidermal ridges [20], were found to be most prominent in the uppermost regions of the digestive zone. Upper pitcher ridging of digestive glands develops earlier, beginning in the elongation phase, and more progressively than in lower pitchers, which begins to ridge only in the inflation phase and are only extensively enveloped in the maturation phase (Figure 5 and Figure 6).

### 2.4. Waxy Scales and Lunate Cells

**Figure 7:**
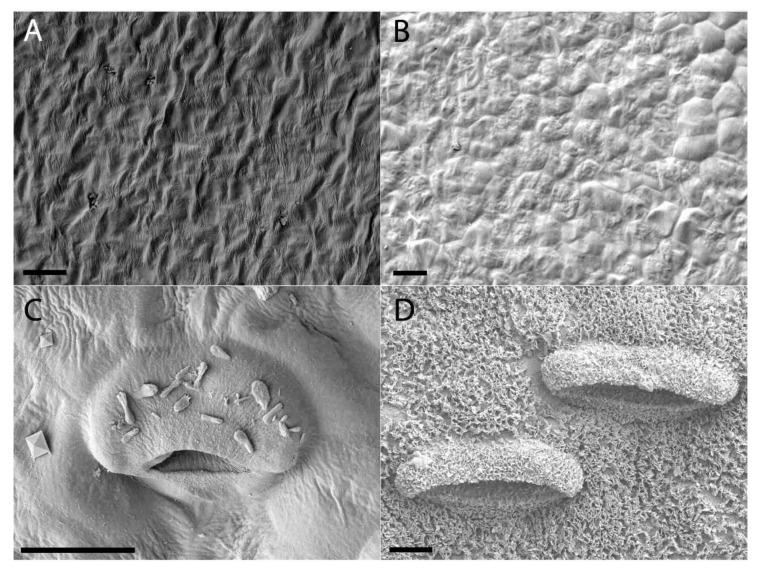
Waxy layer progression and lunate cell development of lower *Nepenthes rafflesiana* pitchers. (**A**) Curvation phase and (**B**) Elongation phase—lunate cells and waxy layer completely absent. (**C**) Inflation phase—lunate cells present and wax crystals on surface. (**D**) Mature phase—significantly waxed surface completely covering the now wider lunate cells and surrounding tissue. Scale bar = 10 µm.

**Figure 8:**
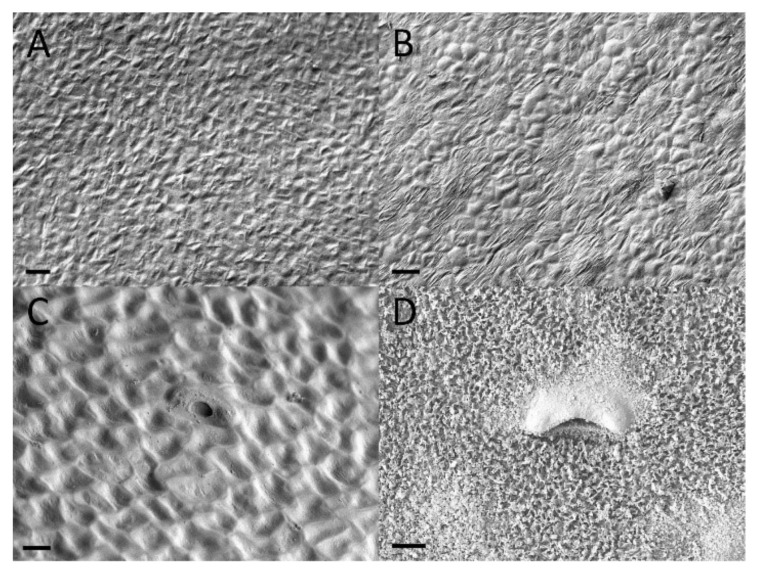
Waxy layer progression and lunate cell development of upper *Nepenthes rafflesiana* pitchers. (**A**) Curvation phase and (**B**) Elongation phase—lunate cells and waxy layer absent. (**C**) Inflation phase—lunate cell begins to develop, no wax yet present. (**D**) Maturation phase—defined lunate cells and wax present. Scale bar = 10 µm.

Lunate cells and the waxy layer that covers this area are typical features of the waxy zone of upper pitchers. Both structures are absent during the first two developmental phases (Figure 7 and Figure 8). Lunate cells can be observed during the Inflation phases, along with a sparse amount of wax. It was only in the mature upper pitcher that large amounts of wax were found to cover the entire waxy zone.

### 2.5. Ontogeny of Upper and Lower Pitchers

**Figure 9:**
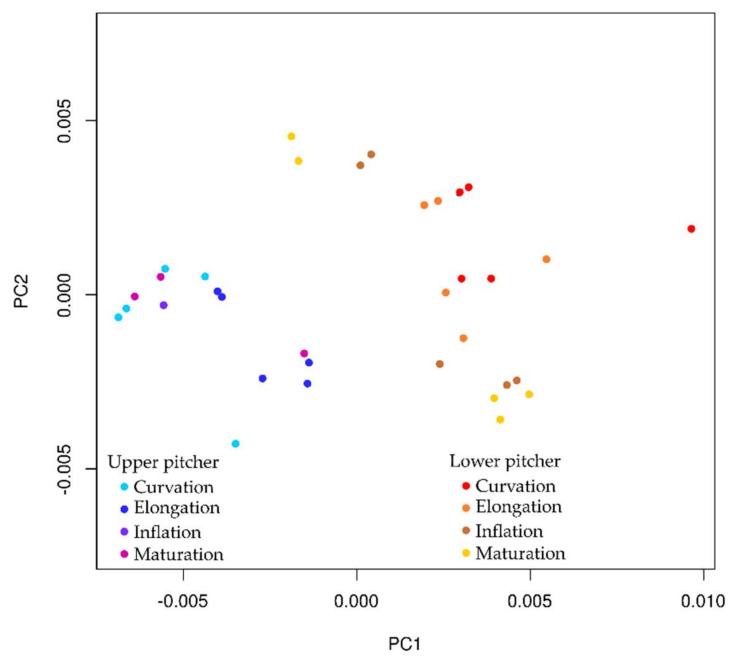
Principal component results for the developmental series of *Nepenthes rafflesiana* upper (left side series in cool colors) and lower (right side series in warm colors) pitchers. Principal component (PC) 1 and 2 separate pitchers from different developmental phases. PC1 accounts for 70.9% of the variance, mainly based on wing length, tendril curvation and pitcher depth. PC2 describes 14.9% of the variance, mainly based on pitcher width.

Three-dimensional models were obtained for the developmental phases of upper and lower pitchers. Our results show that upper and lower pitcher types develop into completely separate shapes from the onset of development in *N. rafflesiana.* Principal Component 1 (PC1) explained 66% of the variance of these 3D models and PC2 explained 21%, so we chose these two variables, as they explained 87% of the variance. Lower and upper pitcher cluster together in the PC plot (Figure 9). Based on these two components, the upper pitcher morphology seems to be entirely separated from lower pitcher morphology.

A linear correlation was observed when PC1 was plotted against pitcher size (as centroid size). Based on regression analysis for PC1 and pitcher size, we could indeed reject H_0_ and conclude that PC1 is dependent on pitcher size (*p* < 0.001). PC2 seems to show a similar correlation with centroid size but this is contradicted by regression analysis (*p* > 0.05).

Principal Component 1 mainly describes changes in height (x) and depth (z) (Figure 9). The most important single landmark in this respect is landmark 9, which describes the position of the lowest point of the curved tendril at the back of the pitcher. Both height (x) and depth (z) coordinates of landmark 9 are major contributors to PC1. Secondly, the length of the wing (x coordinates of landmark 10, 12, 16, and 18) is important when separating the pitchers along with pitcher depth (described by z coordinates of landmarks 7, 15, and 14). In turn, PC2 mainly describes y-coordinates and, thereby, the width of the pitcher (y coordinates of landmarks 2, 3, 5, and 6).

## 3. Discussion

### 3.1. Developmental Phases

In this study, we were able to define four stages of pitcher development based on distinct morphological characteristics. The growth curves of all measured pitchers were found to follow a very similar pattern. Pitcher development was found to be rather constant even under quite variable climate conditions (i.e., light levels experienced in the greenhouses in Leiden during spring, summer, autumn, and winter). However, we found the initiation of new pitchers to be slower during winter months. This suggests that day/night-light hours might be one of the largest contributing factors in pitcher development because temperature remained consistent in the greenhouse. In addition, the moment of tendril curvation and the duration of this first phase are variable, ranging between ten and forty-nine days. From the moment of pitcher elongation, pitcher growth was found to be very similar (Figures S3 and S4). A growth curve of *Nepenthes alata* obtained by Owen and Lennon [20] only showed pitcher size development starting at 40 mm, thereby presumably missing the curvation phase in their graph. This was possibly emitted or avoided on purpose, although not stated explicitly in the methodology because of the variable length of the curvation phase.

We found that after the curvation phase, pitcher size increases gradually during the last three phases of development. We therefore propose to use total pitcher size as an indicator of the developmental phase of a pitcher.

Based on the output of the PCA analysis and the measured variables, we found that wing length, tendril curvation and pitcher depth contribute the most to PC1. PC1 was also found to be dependent on the pitcher size, further strengthening our suggestion that pitcher size can be used as an indicator for developmental phase. PC2 on the other hand is mainly composed of landmarks describing pitcher width. Upper and lower pitchers occupy distinct morphological space based on principal component analysis (Figure 9). Taken together, the four variables described are important features when separating *Nepenthes* pitchers using geometric morphometrics.

### 3.2. Extrafloral Nectary and Peristomal Teeth

Peristomal glands developed early during lower pitcher ontogeny. The presence of vascular tissue in the proximity of the peristomal glands at the inner peristome indicates that these glands are indeed secretory glands, as was previously noted [41]. We found sugar-containing nectar on top of the peristome produced by the peristomal glands at the inside of the peristome-rim. Other carnivorous pitcher plants, including *Cephalotus*, *Darlingtonia*, *Heliamphora*, and *Sarracenia* also have extrafloral nectaries on their pitcher trap surface [42,43]. NMR comparisons of secretions in the lid, with known nectar production [11,44], and the peristome tissue, show that they both contain extrafloral nectaries used to attract animals [8,9,13,45]. It is possible that capillary forces might be involved in transporting this nectar between the peristomal ridges and onto the peristome. Another remarkable finding was that the peristomal glands were hollow. Multiple cells seem to have disintegrated at the site of the cavity, indicating that this intracellular space is formed lysigenously [46]. Vassilyev [41] suggests this cell disintegration occurs because the peristomal glands secrete mucilage prior to lid opening instead of nectar and that the mucilage takes up space specific to where the cells that disintegrate are prior to pitcher opening.

The second type of peristomal gland (found at the underside of the outer peristome) (Figure 4G,H) shows morphological similarities to volatile-producing osmophores [47] and oil glands [48]. We did not test the test for oils or lipid presence associated with these glands, but Di Giusto et al. [11] found that the upper pitcher emit a large quantity of odors, and that the peristomal region is a main source of scent production. Balloon-like structures found in Lamiaceae facilitate volatile evaporation [49]. The fragmented structure of our second peristomal gland (Figure 4G,H) has the appearance of a burst balloon-like structure on the gland.

### 3.3. Digestive Glands

Our finding that digestive glands appear early in development of the upper pitchers might have to do with their importance for insect digestion [22] and their apparent complex structure requiring a relatively long developmental period. Digestive glands are already fully functional from the second developmental phase onwards as we found secretions in the still closed upper pitchers on the surface of the digestive glands (Figure 5 and Figure 6) and nowhere else. This is in accordance with Thornhill et al. [22], who observed digestive glands early in development of *Nepenthes tobaica* and *N. ventricose*, as well as enzyme activity in the digestive fluid, prior to lid opening. Vassilyev [50] similarly found that secretion of digestive enzymes occurred in *Nepenthes khasiana* from digestive glands of still unopened pitchers and before those same glands transitioned to an absorptive function of digested insects once the pitchers opened.

### 3.4. Waxy Scales and Lunate Cells

Waxy scales and lunate cells were not observed in lower pitchers from the first two developmental phases, indicating that lunate cells and wax are formed later in pitcher development. Additional staining with, for instance, phosphotungstic acid, was beyond the scope of this study, but could provide further evidence for the presence of wax. The absence of these structures, however, in these specific pitchers might also have to do with the age of the specific plant at the moment of pitcher formation. Gaume and Di Giusto [17] have shown an ontogenetic loss of wax in juvenile *N. rafflesiana* var. *typica* Beck plants; the waxy layer was found to be reduced in successively produced pitchers on juvenile plants. Ultimately, this results in the formation of pitchers (both lower, as well as upper, pitchers) without a waxy layer. There could be an investment tradeoff for such reduced waxy coatings in pitchers. Highly viscoelastic digestive fluid can be produced as an alternative trapping mechanism in pitchers and was found to be more effective in trapping flying insects than the waxy layer [16]. Flying insects are relatively more abundant higher up in the vegetation, though, so it might be that the plant has to wait until it has reached sufficient height first before it pays off to produce more digestive fluid and less waxy layers in the pitchers.

Gaume and Di Giusto [17] found that the presence of a waxy layer in *N. rafflesiana* var. *typica* had no effect on the number of prey insects found in the lower pitchers. Furthermore, they found that the upper pitchers were completely devoid of such a waxy layer and that lower pitchers only have a waxy layer when they are formed on juvenile plants. Lower pitchers on mature plants are devoid of a waxy layer, which the authors propose occurs because the net benefit of the waxy layer is not as valuable compared to other features, such as digestive fluid viscosity.

### 3.5. Ontogeny of Upper and Lower Pitchers

Pitcher dimorphism is often described as being an ontogenetic process [15,25] because lower pitchers initiate and develop on the plants first before upper pitchers appear. Our results show no overlapping morphology through pitcher development, so paedomorphy (i.e., one form being a state suspended in the developmental process) is not a likely driver of the major phenotypic difference between upper and lower pitcher form. Alternative pathways, such as developmental plasticity within genes caused by epigenetic factors or completely separate genes controlling shape/development could be at play. If temperature or light conditions, for example, were mainly controlling pitcher type, you would expect to see lower pitchers in the place of upper ones (and vice versa) at times, but this is never observed to our knowledge either in the field or in cultivation. In addition, upper and lower pitcher types are often produced simultaneously albeit on different positions on the stem(s), further indicating that more must be at play than the environment alone.

The few studies investigating leaf development in *Nepenthes* from a molecular perspective [51,52] report an increase in proteins during development progresses, but genes responsible for pitcher development have not yet been identified.

## 4. Materials and Methods

### 4.1. Study Species: Nepenthes Rafflesiana

Jack occurs abundantly in the heath forest of Borneo, the southern half of peninsular Malaysia, and more sparsely in Sumatra [53] on nutrient-poor white sands [54]. Plants produce a relatively high number of strongly dimorphic pitchers (Figure 1B,C) each growing season, making this species an ideal candidate for our ontogenetic study. The starting point of development in our study began at the moment of 90° pitcher-tendril curvature as this was the point for which the various morphological features scored were distinguishable for measurement. Lower pitchers were identified as those with a tendril attachment born from the front, or opening side, of the pitcher, facing towards the tendril. They are ellipsoid-shaped and bear ladder-like structures (Figure 1B). The distinguishable upper pitchers have a rear tendril-attachment, are funnel-shaped, and have a prominent, sculpted protrusion on the front side of the pitcher at maturity (Figure 1C). They have their opening facing away from the tendril. Material for the study was harvested from the Hortus botanicus Leiden (7n different clones originating from field collections) at 21 °C with a relative humidity between 40–85% or during fieldwork in Sabah, Borneo, which has similar conditions (Table S1).

### 4.2. 3D Surface Laser Scanning and Landmark-Based Geometric Morphometrics

**Figure 10:**
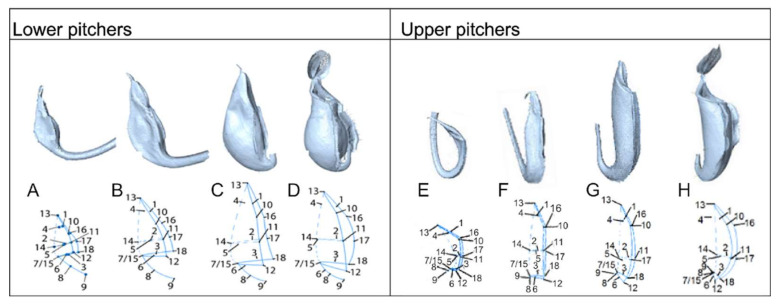
(**A**) Three-dimensional models of *N. rafflesiana* pitchers from all developmental phases and their landmarked coordinates used for morphometric analysis. Lower pitchers of (**A**) Curvation phase, (**B**) Elongation phase, (**C**) Inflation phase, and (**D**) Maturation phase. Upper pitchers (**E**–**H**) are presented with corresponding phases.

Upper and lower pitchers from all developmental stages were three-dimensionally (3D) scanned with a NextEngine 3D scanner HD 2020i (Santa Monica, CA, USA). A total of 35 pitchers were scanned at a maximum resolution of 40,000 points per inch, using the laser triangulation method with multiple laser-stripes sweeping over the pitcher surface. Five pitchers for each developmental phase were either scanned live on the plant or harvested and scanned, with the exception of the upper pitcher inflation (3n) and mature (3n) phases due to scarcity (Table S1). Raw 3D data were auto-aligned, trimmed and refined using ScanStudio HD software version 1.3.2. Eighteen landmarks, which describe the overall pitcher shape (Figure 10 and Figure S8), were applied to the 3D models using Landmark 3.0.0.6. 

We also employed traditional morphometric analysis on the first season of data acquisition that included seven lower pitchers. For this, we measured pitcher length with an electronic digital caliper (0–150 mm) from the hinge of the lid to the lowest point of the curved pitcher base [20] and the width of the pitcher at three different points; directly under the peristome, at the ½ mark of total pitcher length and at the ¾ mark of the total pitcher length (Figures S3 and S4).

### 4.3. Pipeline Created in Galaxy for Statistical Analysis of Pitchers

We wrote several tools in R [55] and the geomorph package [56] to analyze the 3D *Nepenthes* pitcher scans, although the tools could be used for any 3D file that is landmarked. For analysis of the 3D pitcher scans, coordinates from Landmark data files were retrieved followed by a generalized procrustes analysis to minimize differences between pitchers using position, rotation, and scaling [57] from the gpagen function of the geomorph package of R. A principal component analysis (PCA) was performed using the princomp function in R to retrieve principal components explaining the variance in the set and coordinates of the individual pitchers [58]. Principal components (PCs) and centroid sizes of the scanned pitchers against a chosen PC were visualized using the plot function of R. The first 2 PCs explained 87% of the variance, and so were chosen as synthetic shape variables. Additionally, variance was plotted using the barplot function of R.

### 4.4. Scanning Electron Microscopy (SEM)

Freshly collected pitchers were fixed in formaldehyde-acetic acid-alcohol (FAA) (925 mL Ethanol 50%, 50 mL Formalin 37%, 25 mL Acetic Acid 100%) for seven days and then stored in a 50% ethanol solution before SEM preparation. Dissections were made for each developmental phase from the microstructure-containing zones [1,59], which include the areas of the waxy, the digestive and peristomal glands, as illustrated in Figure 1D. Dissections were dehydrated via a series of Ethanol solutions: 50-70-80-96-99.8-99.8%, with ten-minute incubation steps. Dehydrated samples were critical point dried using the Leica EM CPD300. Samples were mounted on SEM-stubs and sputter-coated with a 10 nm layer of Platinum/Palladium-alloy using the Quorum Q150TS. SEM imaging of microstructures was performed at 5.0 kV using the JEOL JSM-7600F SEM. These protocols were developed by the authors.

### 4.5. Light Microscopy (LM)

FAA-fixed peristomal regions of pitchers were dehydrated through a series of ethanol solutions (50-70-90-96-99.8-99.8% for a minimum of eight hours per step. HistoClear replaced ethanol via a gradual increase in HistoClear (25-50-75-100-100%). HistoClear was subsequently replaced by paraplast at 60 °C (33-50-67-100-100%). Samples embedded in Kendall Paraplast Plus (melting point 56–57 °C) were sectioned at 8 µm with an E. Leitz Wetzlar microtome (Wetzlar, Germany), applied on object-glasses, and stained with Etzolds staining solution (stock: 10 mg Basic Fuchsin, 40 mg safranin, 150 mg Astra Blue, 2 mL Acetic Acid filled up to 100 mL with demi-water) for two hours before being washed with demi-water. Paraplast was removed by washing three times for five minutes with HistoClear. DPX Mountant, a standardized mixture of Distyrene, a plasticizer and xylene, was applied between object-glasses and cover-glasses and left to dry overnight. Microscopy slides were observed with an upright Zeiss Axio Imager, M2 Zeiss light microscope (Jena, Germany). Digital images were obtained with a five-megapixel AxioCam MRc 5 (Jena, Germany) and associated AxioVision SE64 Rel. 4.8 software.

### 4.6. Detection of Sugars in Peristomal Fluid

Peristomal fluid (10 µL) was mixed with 2 µL Fehling’s solution A (stock: 3.45 g hydrated copper sulfate and 50 mL demi-water) and 2 µL Fehling’s solution B (stock: 8.25 g sodium potassium, 3.34 g sodium hydroxide and 23.8 mL demi-water) [60]. The mixture was heated for 5 min in a water bath (90 °C). A blue to red color shift indicated the presence of monosaccharides in the peristomal fluid.

Freshly collected pitchers were fixed in liquid nitrogen and ground with an electric blender into powder. A total of 50 mg of this powder was ultrasonicated in 0.75 mL of CH_3_OH-*d_4_* and 0.75 mL of KH_2_PO_4_ buffer in D_2_O (pH 6.0) containing 0.1% (w/w) TMSP for 15 min followed by centrifugation for 13 min at 13,000 rpm. An aliquot of 0.8 mL of the supernatant was transferred into an NMR tube for NMR measurements.

^1^H NMR spectra were recorded at 25 °C on a Bruker 600 MHz AVANCE II NMR spectrometer (Karlsruhe, Germany) operating at a proton NMR frequency of 600.13 MHz equipped with TCI cryoprobe and Z-gradient system. CD3OD was used for internal lock purposes. For 1D-1H NMR spectra, a total of 32,768 data points were recorded covering a spectral window of 9615 Hz. A total of 128 scans of standard one-pulse sequence with 30 degrees flip angle for excitation and presaturation during 2 s relaxation delay were employed with an effective field of cBl = 50 Hz for suppression of the residual H_2_O signal. The protocols for sugar detection follow those developed by the authors and are in line with those used at Naturalis Biodiversity Center (Leiden, The Netherlands).

## 5. Conclusions

In this research, we identified and defined for the first time, four important morphological ontogenetic stages in upper and lower pitchers of *Nepenthes rafflesiana*. Pitcher 3D models of different developmental phases were separated with PCA based on increases in wing length, tendril curvation, pitcher depth (PC1) and pitcher width (PC2). Extrafloral nectariferous peristomal glands developed early during upper pitcher ontogeny in the elongation phase, and we show sugar presence on the tissue from NRM analysis. Functional digestive glands also developed early in pitcher development for both upper and lower pitchers, but enveloped more quickly and progressively in upper pitchers. Lunate cells and a waxy layer were not observed in the first two developmental phases in upper pitchers. Although microstructures and generalized changes occurred in parallel in upper and lower pitcher types, morphometric shapes of upper and lower pitchers are independent ontogenetic processes, with no overlapping quantitative shape. Based on these findings, we conclude that upper pitchers are not a derived state from lower pitchers. Independent developmental programs must have evolved to produce distinctly shaped upper and lower pitchers, possibly to exploit different food sources available near the canopy and forest floor. It would be very interesting to study differences in pitcher morphology between *N. rafflesiana* and other species of *Nepenthes* and possible correlations with diet composition. In addition to this, more knowledge about the genes involved in pitcher initiation and development would move the understanding of pitcher ontogeny forward considerably.

## Supplementary Materials

The following are available online at https://www.mdpi.com/2223-7747/9/11/1603/s1, Figure S1: Nepenthes rafflesiana lower pitcher ontogeny, Figure S2: Nepenthes rafflesiana upper pitcher phases, Figure S3: Pitcher length of seven Nepenthes rafflesiana lower pitchers throughout ontogeny, Figure S4: Width and depth measurements of developing Nepenthes rafflesiana lower pitcher, Figure S5: Sugar region of HNMR spectra of Nepenthes rafflesiana lower pitcher, Figure S6: Overview of digestive gland development of lower Nepenthes rafflesiana pitchers, Figure S7: Overview of digestive gland development of upper Nepenthes rafflesiana pitchers, Figure S8: Landmark category and placement description on Nepenthes rafflesiana pitchers, Figure S9: Overview of waxy layer progression and lunate cell development of lower Nepenthes rafflesiana pitchers, Figure S10: Overview of waxy layer progression and lunate cell development of upper Nepenthes rafflesiana pitchers, Table S1: Specimen information of Nepenthes rafflesiana for Scanning Electron Microscopy and 3D Morphometric analysis.
