# Supplementary material for: Ontogeny and Anatomy of the Dimorphic Pitchers of Nepenthes rafflesiana Jack
Source: Plants (Basel). 2020 Nov 18;9(11):1603. doi: 10.3390/plants9111603 (PMC7698951; doi:10.3390/plants9111603)
Supplement: Supplementary file 1 [file plants-09-01603-s001.pdf]

plants
MDPI

Supplementary Material: Figures S1-S11 & Table S1

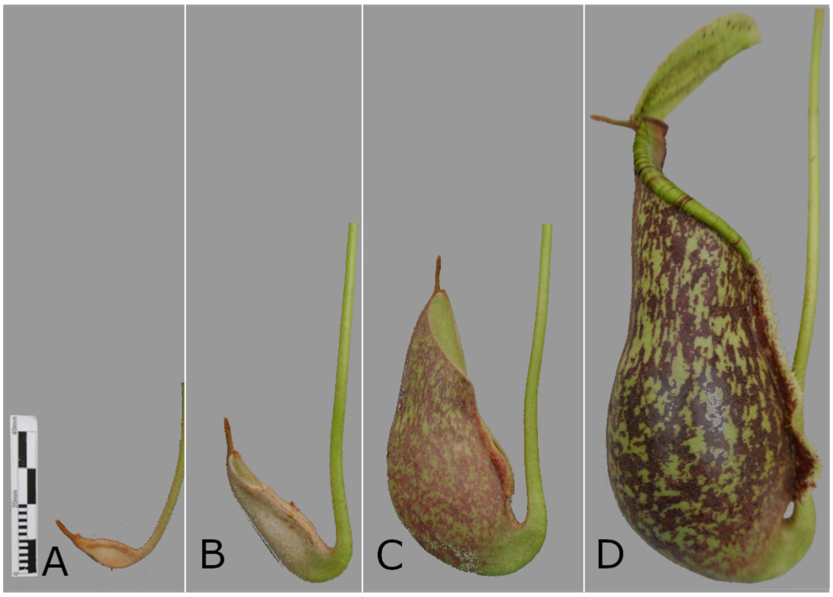

Figure S1. *Nepenthes rafflesiana* lower pitcher ontogeny. (A) Curvation phase, (B) Elongation phase, (C) Inflation phase and (D) Maturation phase. Scale bar = 40 mm.

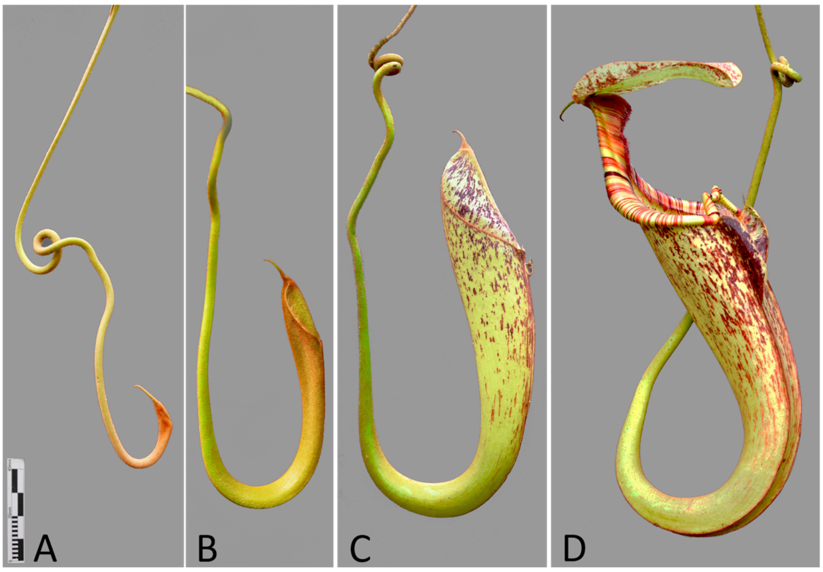

Figure S2. *Nepenthes rafflesiana* upper pitcher phases. (A) Curvation phase, (B) Elongation phase, (C) Inflation phase and (D) Maturation phase. Scale bar = 40 mm. Photographs by Rogier van Vugt.

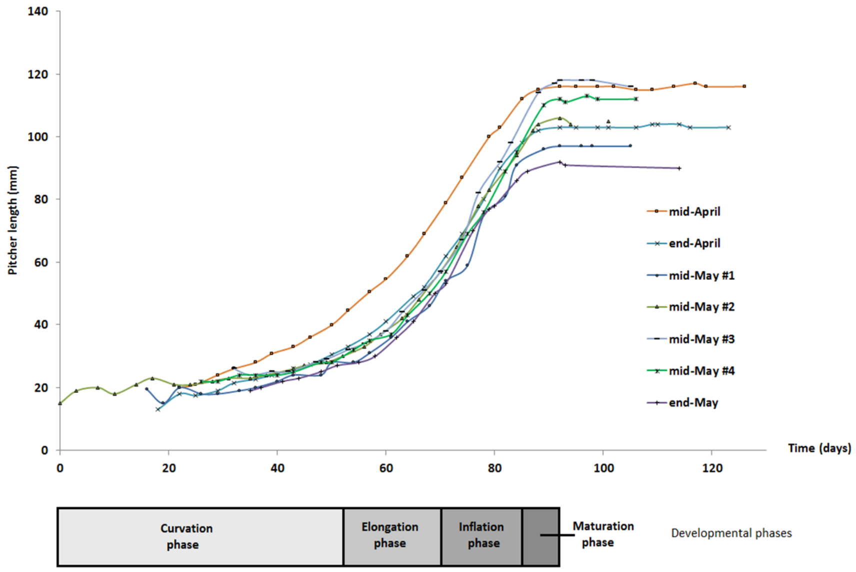

Figure S3. Pitcher length of seven *Nepenthes rafflesiana* lower pitchers throughout development. Pitchers named according to maturation date.

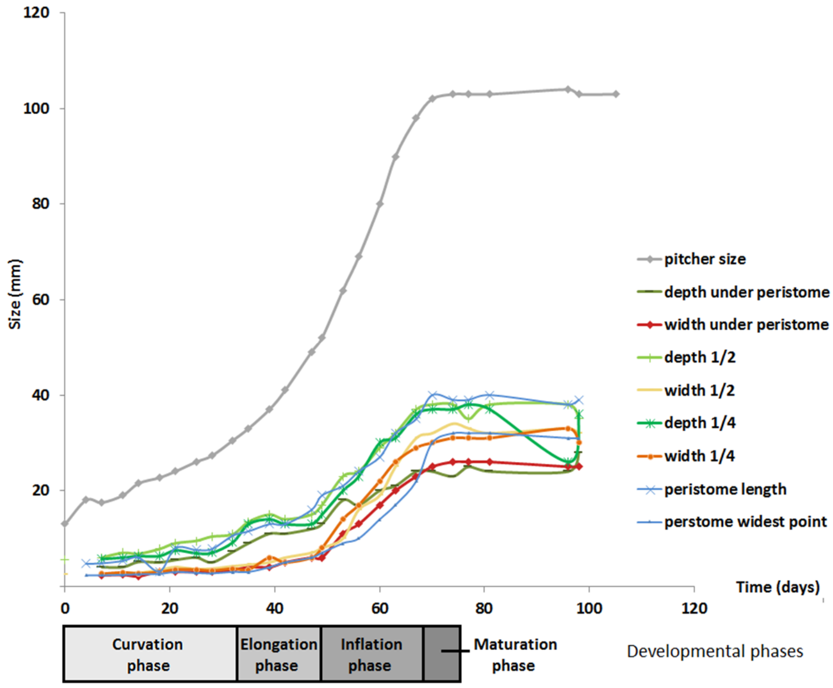

Figure S4. Width and depth measurements of developing *Nepenthes rafflesiana* lower pitcher.

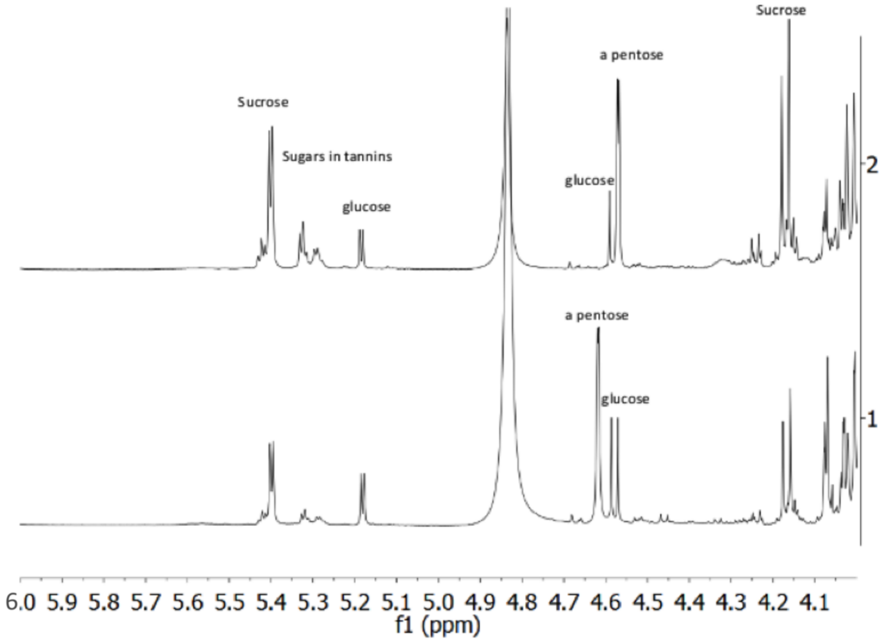

Figure S5. HNMR spectrum chemical shifts (f1 ppm) of *Nepenthes rafflesiana* lower pitcher sugar regions. Pitcher lid (1, bottom) and peristome secretory gland (2, top).

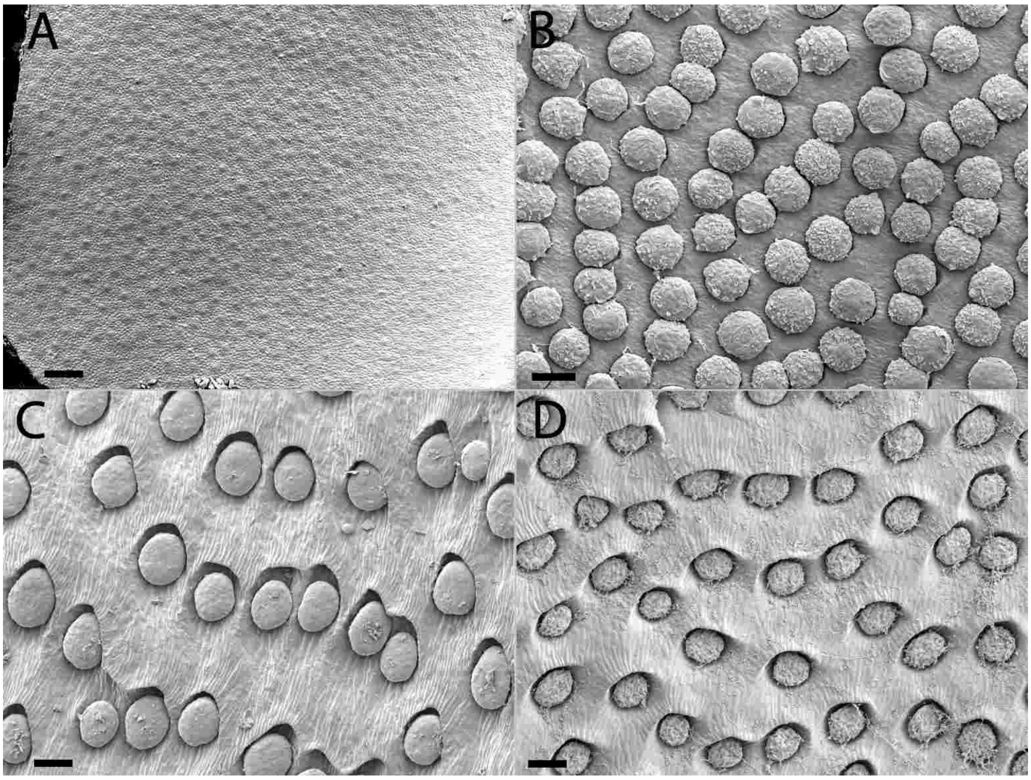

Figure S6. Overview of digestive gland development of lower *Nepenthes rafflesiana* pitchers. Developmental phases include the (A) curvation phase, (B) elongation phase, (C) inflation phase and (D) maturation phase. Scale bar = 100 μm.

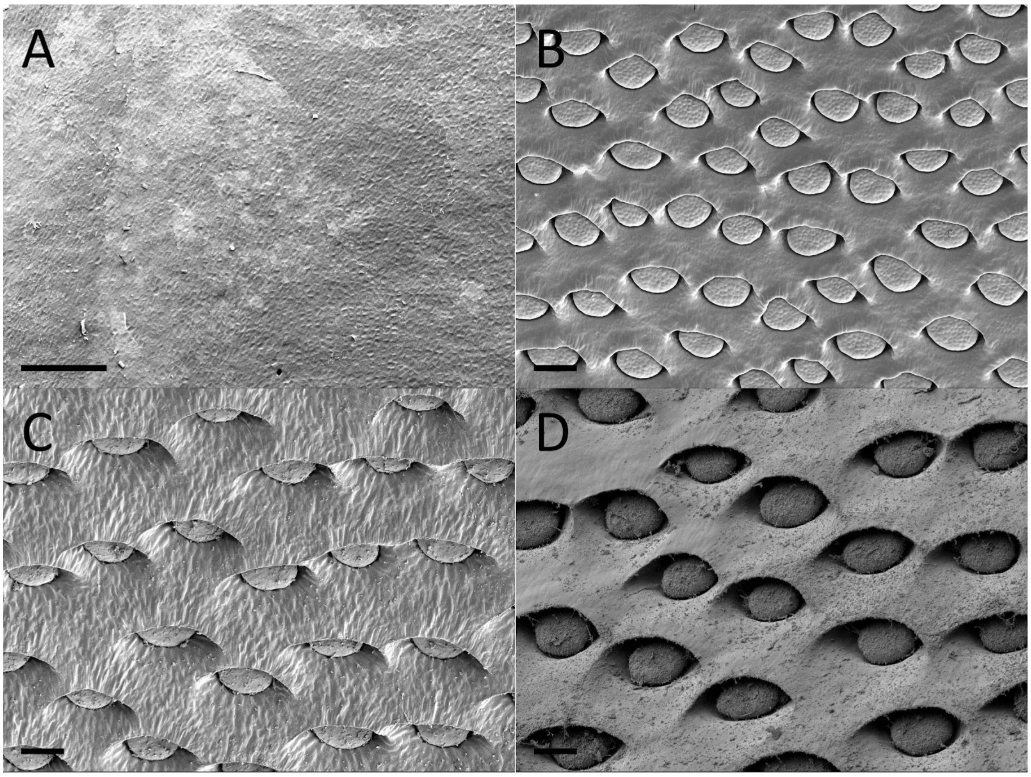

Figure S7. Overview of digestive gland development of upper *Nepenthes rafflesiana* pitchers. Developmental phases include the (A) curvation phase, (B) elongation phase, (C) inflation phase and (D) maturation phase. Scale bar = 100 µm.

Landmark	Type	Description
1	III	Under peristome betw 13 & 16, indicating width (right)
2	III	Halfway between 17 & 14, indicating width (right)
3	III	Halfway between 18 & 15, indicating width (right)
4	III	Under peristome between 13 & 10 indicating width (left)
5	III	Halfway between 11 & 14, indicating width (left)
6	III	Halfway between 12 & 15, indicating width (left)
7	III	Back of pitcher, opposite to lowest point of wing
8	III	Between lowest landmarks (7/15 & 9), on pitcher back
9	II	Lowest point of tendril curvation
10	II	Highest point of pitcher wing (right)
11	III	Middle point of pitcher wing (right)
12	II	Lowest point of pitcher wing (right)
13	II	Directly behind the spur
14	III	Back of pitcher, opposite to middle point of wing
15	III	Back of pitcher, opposite to lowest point of wing
16	II	Highest point of pitcher wing (left)
17	III	Middle point of pitcher wing (left)
18	II	Lowest point of pitcher wing (left)

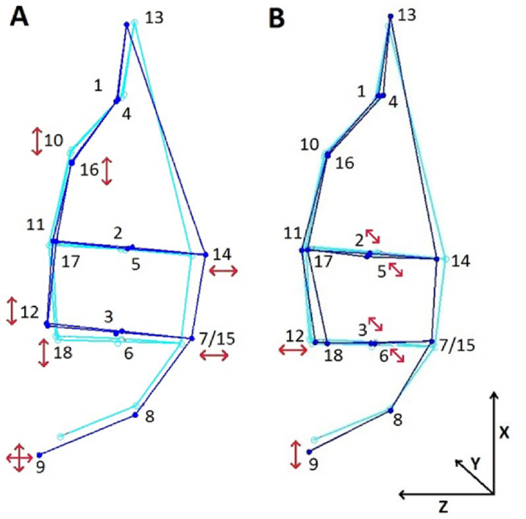

Figure S8. Landmark category and placement description on *Nepenthes rafflesiana* pitchers. Type I landmarks involve the juxtaposition of two types of tissue, type II is a maximum or minimum (top of pitcher, for example) and type III uses type II landmarks as a reference (between top and bottom of pitcher, for example). The right side of the figure shows a side view of pitcher wireframe with landmarks contributing to shape changes. Numbers correspond to landmarks in adjacent table. Red arrows indicate principal component coefficients above 0.200, and the orientation of the arrow indicates the axis (x, y or z). (A) Based on the Principal component coefficients, PC1 encompasses three main shape variables: wing length (landmarks 10,12,16 and 18), tendril curvation (landmark 9) and pitcher depth (landmarks 7/15 and 14). (B) Wireframe of PC2 shows that variance in PC2 is largely described by an increasing pitcher width during development (landmark 2, 3, 5 and 6).

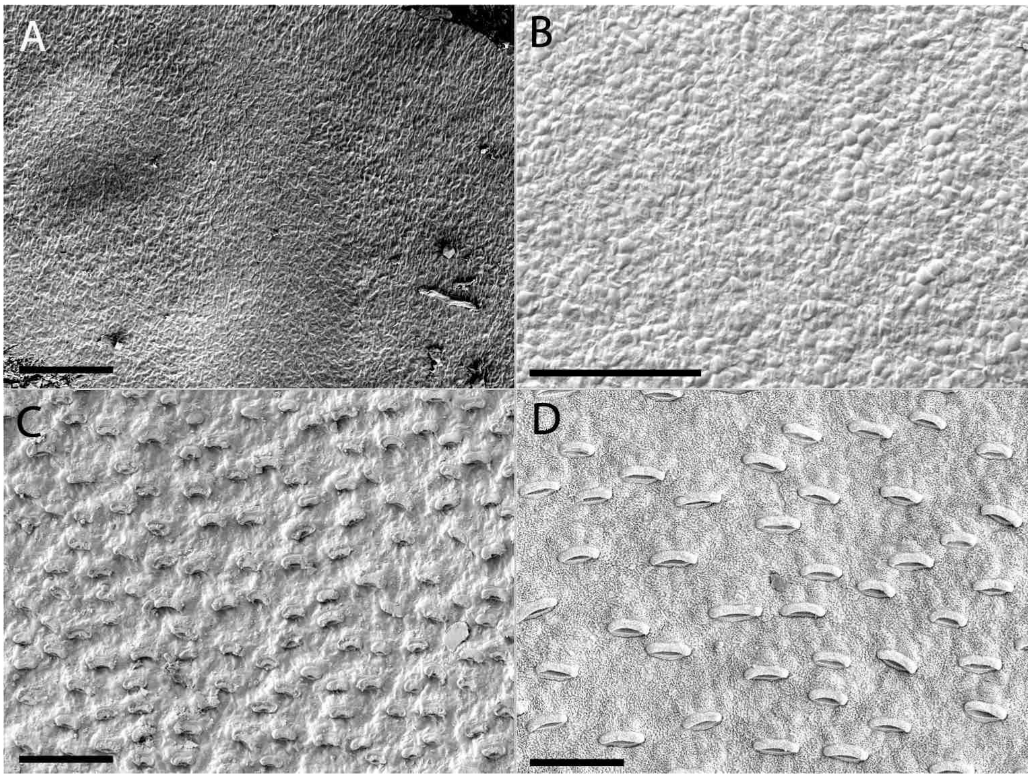

Figure S9. Overview of waxy layer progression and lunate cell development of lower *Nepenthes rafflesiana* pitchers. Developmental phases include the (A) curvation phase, (B) elongation phase, (C) inflation phase and (D) maturation phase. Scale bar = 100 μm.

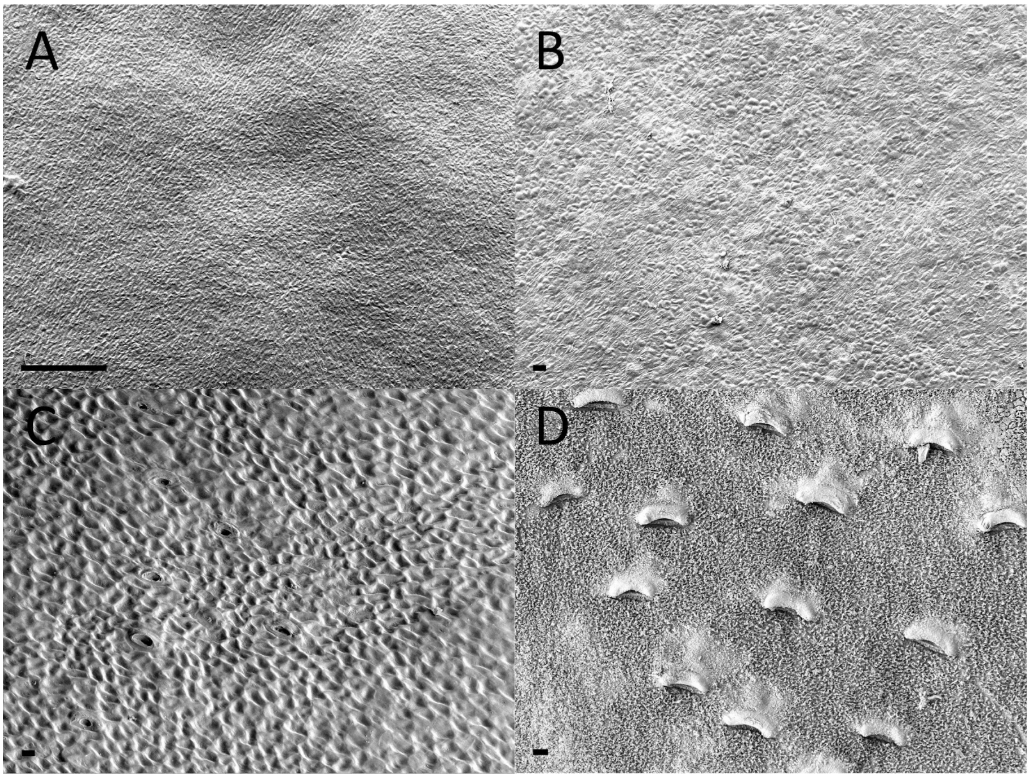

Figure S10. Overview of waxy layer progression and lunate cell development of upper *Nepenthes rafflesiana* pitchers. Developmental phases include the (A) curvation phase, (B) elongation phase, (C) inflation phase and (D) maturation phase. Scale bar = 100 μm.

Supplementary Table 1. Specimen information of *Nepenthes rafflesiana* for Scanning Electron Microscopy and 3D Morphometric analysis. Vouchers for each sample harvested is deposited in the Netherlands (L) and sourced from the *Hortus botanicus* Leiden (HBL) or during fieldwork in Sabah, Borneo (author collection).

Pitcher Type	Voucher	Sample Source
Lower pitcher curvation		
1.1	L.2055339	HBL 817241-23100
1.2	L.2055342	HBL 817241-23100
1.3	L.1026137	HBL 20130488
1.4	L.1026137	HBL 20130488
1.5	L.2055343	HBL 817241-23100
Lower pitcher elongation		
2.1	L.2055342	HBL 817241-23100
2.2	L.2055342	HBL 817241-23100
2.3	L.2055343	HBL 817241-23100
2.4	L.2055339	HBL 817241-23100
2.5	L.2055339	HBL 817241-23100
Lower pitcher inflation		
3.1	L.2055349	HBL A84131-24458
3.2	L.2055344	HBL 84102-24442
3.3	L.2055342	HBL 817241-23100
3.4	L.2055337	HBL 817241
3.5	L.2055347	HBL A84107-23710
Lower pitcher maturation		
4.1	L.2055342	HBL 817241-23100
4.2	L.1026132	HBL 20070071
4.3	L.1026139	HBL 20070071
4.4	L.2055337	HBL 817241-23100
4.5	L.2055339	HBL 817241-23100
Upper pitcher curvation		
5.1	L.1026140	HBL 20130487
5.2	L.1026141	HBL 20130487
5.3	L.1026133	HBL 84107-23710
5.4	L.1026140	HBL 20130487
5.5	L.1026141	HBL 20130487
Upper pitcher elongation		
6.1	L.1026131	HBL A84107-23310
6.2	L.2055340	HBL A84107-23710
6.3	L.2055347	HBL A84107-23710
6.4	L.2055347	HBL A84107-23710
6.5	L.1026139	HBL 20070071

Upper pitcher inflation		
7.1	L.2055336	HBL A84107-23710
7.2	L.1026134	HBL A84107-23310
Upper pitcher maturation		
8.1	L.1026139	HBL 20070071
8.2	L.2055347	HBL A84107-23310
8.3	L.4331617	Author field collection
